# Acute Infection of Viral Pathogens and Their Innate Immune Escape

**DOI:** 10.3389/fmicb.2021.672026

**Published:** 2021-06-22

**Authors:** Kul Raj Rai, Prasha Shrestha, Bincai Yang, Yuhai Chen, Shasha Liu, Mohamed Maarouf, Ji-Long Chen

**Affiliations:** ^1^Key Laboratory of Fujian-Taiwan Animal Pathogen Biology, College of Animal Sciences, Fujian Agriculture and Forestry University, Fuzhou, China; ^2^CAS Key Laboratory of Pathogenic Microbiology and Immunology, Institute of Microbiology, Chinese Academy of Sciences (CAS), Beijing, China

**Keywords:** viral pathogens, acute infection, innate immunity, innate immune escape, non-structural protein

## Abstract

Viral infections can cause rampant disease in human beings, ranging from mild to acute, that can often be fatal unless resolved. An acute viral infection is characterized by sudden or rapid onset of disease, which can be resolved quickly by robust innate immune responses exerted by the host or, instead, may kill the host. Immediately after viral infection, elements of innate immunity, such as physical barriers, various phagocytic cells, group of cytokines, interferons (IFNs), and IFN-stimulated genes, provide the first line of defense for viral clearance. Innate immunity not only plays a critical role in rapid viral clearance but can also lead to disease progression through immune-mediated host tissue injury. Although elements of antiviral innate immunity are armed to counter the viral invasion, viruses have evolved various strategies to escape host immune surveillance to establish successful infections. Understanding complex mechanisms underlying the interaction between viruses and host’s innate immune system would help develop rational treatment strategies for acute viral infectious diseases. In this review, we discuss the pathogenesis of acute infections caused by viral pathogens and highlight broad immune escape strategies exhibited by viruses.

## Introduction

Viral pathogens are infectious particles containing either DNA or RNA as their genome. A large number of viruses belonging to various families cause rampant disease in human beings, ranging from mild and self-limiting to acute fatal diseases (Herrington et al., 2015; Keighley et al., 2015; Jacob et al., 2020). Various viral families, such as *Filoviridae*, *Arenaviridae*, *Bunyaviridae*, *Paramyxoviridae*, *Coronaviridae*, *Orthomyxoviridae*, *Flaviviridae*, *Togaviridae*, *Hepeviridae*, and so forth, infect humans and/or animals. Unfortunately, emerging and re-emerging viral pathogens often cause catastrophic pandemics that may take millions of human lives. For example, the most devastating “Spanish flu” pandemic in 1918 took over 50 million lives. The subsequent emergence of flu pandemics, such as “Asian flu” and “Hong Kong flu” in 1957 and 1968, respectively, killed about three million people (Salomon and Webster, 2009). During 2002 and 2003, a novel severe acute respiratory syndrome coronavirus (SARS-CoV) infected over 8,000 people, causing 774 deaths in 27 countries (World Health Organization, 2018). A new virulent Influenza A Virus (IAV) H1N1 strain (H1N1pdm09) emerged in 2009 that killed ∼151,700–575,400 people worldwide (Centre for Disease Control and Prevention (CDC), 2012). A new avian IAV strain (H7N9), “Bird flu,” and the Middle East respiratory syndrome (MERS)-CoV in 2013 also emerged (To et al., 2013). Some viruses re-emerged after a number of years, such as the re-emergence of the Ebola virus (EBOV) in 2014 (Shen et al., 2015), resurgences of the Zika virus (ZIKV) in 2015 and 2016 (Shuaib et al., 2016), and so forth (Chauhan et al., 2020; Guo, 2020). The ongoing pandemic caused by SARS-CoV-2 has already ravaged humanity and is still on the rise across the globe. Health challenges and economic consequences caused by the ongoing Covid-19 pandemic are potentially devastating and may remain an enduring puzzle. Therefore, a better understanding of the complex underlying mechanisms of viral pathogenesis caused by acute infections is truly important to the human community.

Innate immunity is a critical first line of defense against viral invasion. A well-specialized immune system consisting of distinct physical and chemical barriers, such as mucosal surfaces, skin, and their secretions, counter against viral invasion during viral entry into the host. Viruses are further sensed by various pattern recognition receptors (PRRs) after their entry, which leads to the activation of innate immune signaling pathways that control the production of interferons (IFNs), pro-inflammatory cytokines, and chemokines. Type I and III IFNs produced by various types of cells stimulate the expression of hundreds of genes, collectively known as IFN-stimulated genes (ISGs), which prime cells into an antiviral state (Iwasaki and Pillai, 2014; Chen et al., 2018). Secreted pro-inflammatory cytokines cause local and systemic inflammation. Chemokines produced at the site of infection may recruit additional immune cells, including neutrophils, monocytes, and natural killer cells (Christensen and Thomsen, 2009; Chen et al., 2018). Then, virus-infected cells could be targeted by immune cells, which mediates viral clearance (Iwasaki and Pillai, 2014). An acute viral infection is characterized by sudden or rapid onset of disease that may be fatal. Viral clearance during acute infection correlates with rapid induction of innate immunity, especially induction of ISGs, and subsequent induction of adaptive immune responses (Heim and Thimme, 2014). On the other hand, viruses are ever-evolving and can emerge and re-emerge into newer/novel virulent strains. The emergence of viral variants with increased adaptability and/or virulence indicates that viruses are acquiring new strain-specific mechanisms of immune escape. Viruses can develop multiple tactics to subvert innate immune surveillance and escape detection by innate immune sensors, leading to suppression of PRRs and their downstream signaling cascades to establish a successful infection. For instance, non-structural proteins of influenza and members of *Flaviviridae* viruses deploy numerous tactics to potently inhibit type I IFN signaling (Marc, 2014; Li et al., 2015; Chen et al., 2017). SARS-CoV-2 deregulates type I IFN responses through multiple mechanisms (Acharya et al., 2020). ZIKV circumvents host innate immunity by targeting the adaptor proteins MAVS and MITA (Li et al., 2019). Enteroviruses brilliantly exploit their viral proteinase (3C^pro^ and 2A^pro^) to cleave PRRs (RIG-I, MDA5) and immune adaptor molecules (MAVS and TRIF), and thereby dampen the production of type I and III IFNs (Mukherjee et al., 2011; Feng et al., 2014; Lind et al., 2016). Cooperation among non-structural proteins (NS1, NS4B, and NS2B3) of ZIKV appeared to attenuate antiviral immunity (Wu et al., 2017). In this review, we describe the pathogenesis of acute viral infections in relation to host innate immunity and discuss how viruses escape innate immune surveillance.

## Pathogenesis of Acute Viral Infections

Being obligated intracellular parasitic infectious particles, viruses replicate only inside their specific host cell or tissue. For viruses to cause diseases, they must first infect their specific host, replicate efficiently within the host, and damage targeted tissues. Viral pathogenesis is complex and disease outcomes are determined by multiple factors (MacLachlan and Dubovi, 2017). Viruses rely on numerous host factors (determinants) to replicate efficiently in the host to cause disease (MacLachlan and Dubovi, 2017; Long et al., 2019; Gerold et al., 2020). Some hosts are highly susceptible to viral infection, while some are resistant. Differential host susceptibility to viral infection and disease progression depends on both viral infectivity (virulence) and host responses (Long et al., 2019; Gerold et al., 2020). Of those host responses, innate immunity plays a critical role in viral clearance and disease progression. During viral infection, various factors including delicate and dynamic equilibrium between pro-inflammatory and anti-inflammatory responses, immune cell activation and deactivation, and IFNs upregulation and IFN-reversion to the baseline, play important roles in viral pathogenesis and progression of disease (Virgin et al., 2009; Osburn et al., 2013; Maarouf et al., 2018; Blanco-Melo et al., 2020). For example, an imbalanced response that is characterized by low levels of type I and III IFNs juxtaposed to elevated chemokines and high expression of IL-6 to SARS-CoV-2 drives the development of COVID-19 (Blanco-Melo et al., 2020).

An acute viral infection can be resolved quickly by immune responses exerted by the host. For example, acute Hepatitis B Virus (HBV) infection can be spontaneously resolved in more than 90% of infected adults, although HBV can sometimes result in chronic persistent infection (Shin et al., 2016). The inflammatory response must be well-regulated in the course of viral clearance. However, excessive inflammatory responses can be lethal. Elevated levels of a broad array of pro-inflammatory cytokines and chemokines have been observed in diseases caused by various acute viral infections, such as EBOV disease, severe lung injury by infection of IAV, respiratory syncytial virus (RSV), and SARS-CoV-2 (Virgin et al., 2009; Shin et al., 2016; Troy and Bosco, 2016; Maarouf et al., 2018; Blanco-Melo et al., 2020). Acute respiratory infections are the leading cause of global disease (Troy and Bosco, 2016). Immune responses and disease outcomes in acute HAV (Hepatitis A Virus), HBV, and HCV infections have been previously described (Shin et al., 2016). Clinical manifestations, etiology, and outcome of various viral diseases caused by a large group of numerous viral infections have been described/reviewed elsewhere (Whitton et al., 2005; Ascenzi et al., 2008; Gould and Solomon, 2008; Ramos-Casals et al., 2008; Rojek and Kunz, 2008; Newton et al., 2016; Troy and Bosco, 2016; Zuberbier et al., 2018).

## Innate Immunity

### Innate Detection of Viral Infections

Immediately after viral infection, elements of innate immunity, such as physical barriers, various phagocytic cells, group of cytokines, IFNs, and IFN-stimulated genes, provide the first line of defense for viral clearance. Physical barriers, such as mucosa, skin, mucous membranes, tears, earwax, mucus, and stomach acid provide preliminary defense against invading viruses (Sanders et al., 2011; Doran et al., 2013; Chen et al., 2018). If viral invaders breach physical barriers, viruses are detected/sensed by germline-encoded immune molecules PRRs (Lazear et al., 2013; Iwasaki and Pillai, 2014; Chen et al., 2018; Chiang and Liu, 2019). Toll-like receptors (TLRs), such as TLR2/3/4/7/8/9 are important immune detectors involved in the induction of innate immunity (Kawai and Akira, 2010). TLR2 and 4 detect extracellular viral proteins at the cell surface. Intracellular viral dsRNA, ssRNA, and DNA are recognized by TLR3, TLR7, TLR8, and TLR9, at intracellular endosomal compartments during endocytosis and autophagy (Kawai and Akira, 2010; Maarouf et al., 2018). Retinoic acid-inducible gene I (RIG-I) like receptors, including RIG-I and melanoma differentiation-associated protein 5 (MDA5), are key intracellular sensors of viral RNA (Kawai and Akira, 2010; Morgan Brisse, 2019). RIG-I plays an important role in the detection of several viruses, such as orthomyxoviruses, rhabdoviruses, and arenaviruses, and MDA5 preferentially detects picornaviruses. Additionally, many other viruses, such as flaviviruses, paramyxoviruses, reoviruses, and others are also detected by both RIG-I and MDA5 (Morgan Brisse, 2019). Cumulative pieces of evidence have also shown that paramyxoviruses, some flaviviruses [for example, Dengue Virus (DENV) and West Nile Virus (WNV)], and reoviruses, may be sensed by both RIG-I and MDA5 (Kawai and Akira, 2010; Goubau et al., 2013; Chan and Gack, 2016; Morgan Brisse, 2019). Well-known viral pathogens responsible for acute respiratory infections, such as SARS-CoV, SARS-CoV-2, and MERS-CoV are detected by endosomal PRRs, including TLR3 and 7, and/or cytoplasmic sensors, such as RIG-I and MDA5 (Felsenstein et al., 2020; Liu et al., 2020). NOD-like receptors (NLRs) are a large family of intracellular PRRs. Members of the NLR family assemble into large multiprotein complexes, termed inflammasomes. Many viruses, including rotavirus, Sendai Virus (SeV), and IAV, can activate inflammasomes (Shrivastava et al., 2016). Cyclic-GMP-AMP (cGAMP) synthase (cGAS) and gamma-IFN-inducible protein 16 (IFI16) are well characterized as intracellular detectors of DNA viruses and viral DNA intermediates (Koyama et al., 2008; Ma et al., 2018).

### Innate Immune Signaling

**FIGURE 1:**
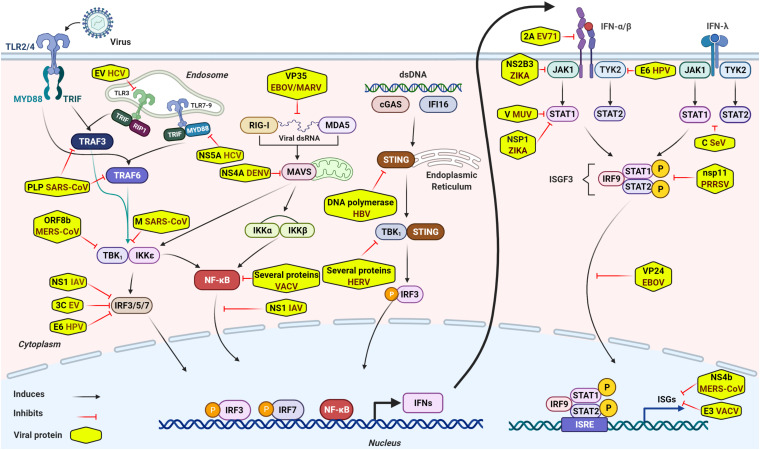
General overview of intracellular innate immune signaling and some representative viral immune escape mechanisms. Sensing virus by PRRs initiates innate immune signaling through the hierarchical activation of PRRs family-specific adaptor proteins (TRIF, MAVS, STING, MYD88, and so forth) to activate transcriptional factors, such as IRF3/5/7, NF-kB, and others. Activated transcriptional factors translocate into nucleus and induce robust expression of IFNs. Secreted IFNs bind to their respective receptors and activate JAK-STAT signaling and form a transcriptional factor called ISGF3. ISGF3, then, translocates into nucleus to induce expression of numerous antiviral effectors (ISGs) to impede viral infection. Although antiviral innate immunity consists of well-equipped arsenals to impede viral infection and invasion, viruses circumvent or escape from these antiviral arsenals to establish successful infection through several mechanisms. Of these escape mechanisms, viral components inhibit innate immune signaling by diversified tactics, such as interacting directly or indirectly with crucial innate elements, targeting and cleaving adaptor proteins involved in innate immune signaling or interference of IFN signaling, degradation of JAK/STAT components, and so forth. Some representative viral immune escape tactics are shown in the Figure 1.

Innate immune signaling is initiated by sensing specific viral components, called pathogen associate molecular patterns (PAMPs), such as viral dsRNA, ssRNA, DNA, transcription products, and other viral components including replication intermediates. The sense of PAMPs by PRRs leads to the activation of downstream molecules including mitochondrial antiviral signaling protein (MAVS), stimulator of IFN genes (STING) or MYD88, and transcription factors, such as interferon regulatory factors (IRF3/5/7), NF-kB, AP1, and so forth (Koyama et al., 2008; Ishikawa et al., 2009; Kawai and Akira, 2010; Rathinam and Fitzgerald, 2011; Jensen and Thomsen, 2012; Goubau et al., 2013; Lazear et al., 2013; Iwasaki and Pillai, 2014; Goraya et al., 2015; Chan and Gack, 2016; Shrivastava et al., 2016; Chen et al., 2018; Ma et al., 2018; Chiang and Liu, 2019; Morgan Brisse, 2019; Felsenstein et al., 2020; Liu et al., 2020). The PRR-mediated signaling pathways ultimately lead to the secretion of numerous antiviral molecules, including type I and type III IFNs, and other pro-inflammatory cytokines and chemokines (Koyama et al., 2008; Ishikawa et al., 2009; Kawai and Akira, 2010; Rathinam and Fitzgerald, 2011; Jensen and Thomsen, 2012; Goubau et al., 2013; Lazear et al., 2013; Iwasaki and Pillai, 2014; Goraya et al., 2015; Chan and Gack, 2016; Shrivastava et al., 2016; Chen et al., 2018; Ma et al., 2018; Chiang and Liu, 2019; Morgan Brisse, 2019; Felsenstein et al., 2020; Liu et al., 2020). The secreted IFNs bind to their respective receptors and activate Janus protein tyrosine kinase-signal transducer and activator of transcription (JAK-STAT) pathway (Majoros et al., 2017) that results in the production of hundreds of downstream antiviral ISGs, such as MX1, ISG15, IFITM3, and viperin, which establish an antiviral state to impede virus infection (Iwasaki and Pillai, 2014; Schoggins, 2014; Figure 1). Recently, activation of the innate immunity independently of cytokine signaling through RIG-I/MAVS/Syk/STAT1 pathway at the early stage of viral infection has also been reported (Liu et al., 2021). The mechanistic basis of innate immune signaling induced by several viral infections has been extensively reviewed elsewhere (see review papers Koyama et al., 2008; Ishikawa et al., 2009; Kawai and Akira, 2010; Rathinam and Fitzgerald, 2011; Jensen and Thomsen, 2012; Goubau et al., 2013; Lazear et al., 2013; Iwasaki and Pillai, 2014; Schoggins, 2014; Goraya et al., 2015; Chan and Gack, 2016; Shrivastava et al., 2016; Majoros et al., 2017; Chen et al., 2018; Ma et al., 2018; Chiang and Liu, 2019; Morgan Brisse, 2019; Felsenstein et al., 2020; Liu et al., 2020, 2021).

### Roles of Interferons in Antiviral Responses

Interferons (IFNs), a family of cytokines, are critical elements of innate immunity responsible for rapid and efficient viral clearance (Fensterl and Sen, 2009). Virtually all nucleated cells could express IFNs during viral infection and IFN production is the key antiviral process of innate immunity during viral infection. Type I (IFN-α/β) and Type III IFNs are principal IFNs produced during viral infection as a key part of the innate immune response. The typical feature of IFNs is to induce upregulation of a wide array of intracellular antiviral effectors called ISGs through JAK-STAT signal pathway (Fensterl and Sen, 2009; Schoggins, 2014; Majoros et al., 2017; Paul et al., 2018). Type I and type III IFNs bind to their respective receptors on the infected cell and neighboring cells, which leads to activation of JAK-STAT pathway and nuclear translocation of STAT1/STAT2/IRF9 (ISGF3) (Reviewed in Fensterl and Sen, 2009; Schoggins, 2014; Paul et al., 2018) and results in induction of numerous ISGs, such as Mx proteins, ISG15, protein kinase PKR, 2’-5’-oligoadenylate synthetases (OAS), ribonuclease L (RNaseL), IFN-inducible dsRNA-dependent protein kinase (PKR), adenosine deaminase RNA-specific and apolipoprotein B mRNA-editing enzyme, catalytic polypeptide 3, and others to establish an antiviral state (Iwasaki and Pillai, 2014; Chen et al., 2018). Of note, some ISGs, such as OAS and PKR, are further activated by dsRNA, which, in turn, inhibit viral replication by various mechanisms (Ishikawa et al., 2009; Iwasaki and Pillai, 2014; Chen et al., 2018). Additionally, IFNs may also exert immunomodulatory functions that affect cell migration, cross-presentation, CD4^+^ T cell stimulation or CD8^+^ T cell clonal expansion, and B cell activation, and enhance antiviral humoral responses (Iwasaki and Pillai, 2014). Thus, before the effective adaptive immunity is initiated, IFN-mediated innate immune response plays critical roles in eliminating virus invasion (Iwasaki and Pillai, 2014). Although viral infection induces the rapid expression of IFNs and antiviral effectors, at the same time, viral components can suppress IFNs signaling. Theoretically, virus-induced robust IFNs production, IFN-reversion to baseline by viral antagonisms, and optimal ISGs expression could establish a steady, delicate, and dynamic equilibrium. However, destruction of such a steady state, particularly in acute viral infection, by hyper-production of IFNs and hyper-effective immune evasion is the primary cause of viral pathogenesis (Maarouf et al., 2018; Blanco-Melo et al., 2020). Both hyper-production of IFNs and hyper-effective immune evasion are disadvantageous to the host. Imbalanced levels of IFNs expression and differential ISGs production differ across type of viruses, which also determines the viral pathogenesis. For instance, among three Hepatitis (A, B, and C) viruses, HCV infection induces the robust expression of a large number of ISGs, whereas HAV infection minimally induces ISG expression and HBV infection might not induce ISG expression (Shin et al., 2016). Moreover, viruses are ever-evolving to escape away from innate immunity, particularly at acute infection. For example, IFITM is a critical antiviral ISG against several viruses including Human Immunodeficiency Virus (HIV-1), however, transmitted founder HIV-1viruses are uniquely IFITM resistant, a property that is lost during chronic infection. This is in part due to escape mutations acquired in response to autologous neutralizing responses (Foster et al., 2016).

## Cytokine Storm Caused by Acute Viral Infection

Optimal activation of innate immunity in the course of viral infection is very important for viral clearance. However, an acute viral infection usually causes over-activation of innate immunity. Such over-activation may induce robust and hyper-production of IFNs, proinflammatory and anti-inflammatory cytokine, and chemokines, including excessive secretion of TNF-α, vascular endothelial growth factors (VEGF-A), IL-1, IL-6, IL-10, IL-8, CCL2, CXCL10, and so on, leading to cytokine storms (Wauquier et al., 2010; Liu et al., 2016; Srikiatkhachorn et al., 2017; Teijaro, 2017; Blanco-Melo et al., 2020; Gerges Harb et al., 2020; Mahmudpour et al., 2020; Vabret et al., 2020). Cytokine storms released during acute viral infection can result in single or multiple organ damage and even death (Wauquier et al., 2010; Liu et al., 2016; Srikiatkhachorn et al., 2017; Teijaro, 2017; Maarouf et al., 2018; Blanco-Melo et al., 2020; Gerges Harb et al., 2020; Mahmudpour et al., 2020; Vabret et al., 2020). For example, in COVID-19, the cytokine storm is an important factor leading to the death of many patients (Mahmudpour et al., 2020; Vabret et al., 2020). Cytokine storms caused by acute viral infection, such as influenza virus, coronavirus, Ebola virus, dengue virus, and so forth have been extensively reviewed elsewhere (Wauquier et al., 2010; Liu et al., 2016; Srikiatkhachorn et al., 2017; Teijaro, 2017; Gerges Harb et al., 2020; Mahmudpour et al., 2020; Vabret et al., 2020).

## Viral Innate Immune Escape Strategies

Nonetheless, the host is well-equipped with innate antiviral arsenals to eliminate invading viral pathogens; viruses evolved strategies to escape innate immune surveillance. At the early stage of viral entry into the host, viruses breach hosts’ physical barriers by various ways. Upon breaching physical barriers, viruses exploit diverse mechanisms to inhibit the activation of PRRs and their downstream signaling cascades, such as concealing their PAMPs, interacting directly or indirectly with crucial innate elements, such as PRRs, transcriptional factors, targeting and cleaving adaptor proteins involved in innate immune signaling or interference of IFN signaling, degradation of JAK/STAT components, and so forth (Table 1). A broad mechanism of viral innate immune escape tactics is discussed below.

**TABLE 1:** Representative immune escape strategies.

Viruses	Innate elements and mechanisms	References
**Penetrating physical barrier**		
Several viruses, such as Coxsackie, swine vesicular disease virus, adenovirus, reovirus, and others	Breach mucosa by targeting proteins of the apical junctional complex	Gonzalez-Mariscal et al., 2009
ZIKV, DENV, and WNV	Breach skin barrier by infecting permissive cells	Garcia et al., 2017
HIV/SIV	Penetrate physical barrier in multiple ways	Keele and Estes, 2011
**Interference with PRRs signaling**		
HCV	Extracellular vesicles mask HCV dsRNA to reduce activation of TLR3.	Grünvogel et al., 2018
SARS-CoV	Viral Papain-Like Protease antagonize the TLR7 signaling through removing Lys63-Linked polyubiquitination of TNF receptor-associated factors (TRAF3 and TRAF6)	Li et al., 2016
Marburg virus (MARV) and EBOV	VP35 protein binds to viral dsRNA genomes to inhibit viral sensing by RIG-1 and MDA-5.	Ramanan et al., 2012
HBV	Escape from cGAS sensing by the packaging of the genome into the viral capsid	Verrier et al., 2018
Vaccinia virus (VACV) and IAV	E3L and NS1 proteins of respective viruses sequester viral dsRNA to escape away from sensing by PRRs	Chang et al., 1992; Hatada and Fukuda, 1992
Enterovirus (EV)	Viral proteinases 3C^pro^ and 2A^pro^ counteracts PRRs signaling by targeting RIG-I and MDA5, respectively.	Feng et al., 2014; Lind et al., 2016
HCV	NS5A protein inhibits TLR signaling by associating with MYD88	Abe et al., 2007
VACV	A46R targets multiple Toll-like-interleukin-1 receptor adaptors	Stack et al., 2005
**Inhibition of transcriptional factors IRF3/7, NF-kB, and AP1**		
SARS-CoV-2	Suppresses the activation of TRAF3 and TRAF3 and thereby inhibit IRF3/7 and NF-kB activation	Liu et al., 2020
MERS-CoV	Accessory protein ORF8b suppresses MDA5 and TBK1 medicated NF-κB signaling and M protein suppresses type TBK1-dependent phosphorylation of IRF3	Lui et al., 2016; Lee et al., 2019
IAV	NS1 protein inhibits nuclear translocation of IRFs and NF-kB	Wang et al., 2000
HPV	Interfere in critical ubiquitination events upstream of IRF-3 and NFκB by upregulating the cellular deubiquitinase UCHL1	Karim et al., 2013
HCV	NS5A viral protein inhibits nuclear translocation of AP-1 by interacting with Grb2	Macdonald et al., 2003
VACV	Several viral proteins, such as A46, A49, A52, and others inhibit NF-kB activation by multiple mechanisms.	Smith et al., 2013
EV	Viral 3C proteases cleavs IRF7	Lei et al., 2013
SARS-CoV	Viral M protein inhibits IRF3/7 activation targeting TBK1/IKKε	Siu et al., 2009
EBOV	VP35 protein inhibits IRF3 phosphorylation and subsequent dimerization	Basler et al., 2003
Human papilloma virus 16	Viral E6 oncoprotein binds to IRF3 and inhibits its transcriptional activity	Ronco et al., 1998
**Interference of JAK-STAT signaling**		
HPV 18	Viral E6 oncoprotein binds with Tyk2 and impairs JAK-STAT activation	Li et al., 1999
Mumps virus (MUV)	V protein induces degradation of STAT-1 and STAT-3	Ulane et al., 2003
HSV-1	Inhibits JAK-STAT signaling by inducing SOCS3	Yokota et al., 2004
SeV	C protein inhibits the phosphorylation of STAT1 and STAT2	Oda et al., 2017
ZIKV, DENV	Induce STAT2 degradation	Morrison et al., 2013; Grant et al., 2016
ZIKV	NS2B3 protein promotes the degradation of Jak1	Wu et al., 2017
EBOV	EBOV VP24 binds to the α5 and α6 subunits of importin, which are the essential components of the nuclear transporter, to block the nuclear translocation of phosphorylated STAT1	Shabman et al., 2011
Rotavirus	NSP1 protein inhibits STAT1 activation	Sen et al., 2014
Nipah and Hendra virus	Nucleoproteins inhibit the nuclear accumulation of STAT1 and STAT2 and interfere with their complex formation	Sugai et al., 2017
Parainfluenza virus type 1	C protein binds and retains STAT1 in perinuclear aggregates at the late endosome	Schomacker et al., 2017
Porcine reproductive and respiratory syndrome virus (PRRSV)	Nsp11 protein interacts with IRF9 and formation and nuclear translocation of the transcription factor complex IFN-stimulated gene factor 3 (ISGF3)	Wang et al., 2019
**Antagonizing ISGs**		
VACV	Viral E3 protein interacts with human and mouse ISG15	Eduardo-Correia et al., 2014
MERS-CoV	NS4b proteins cause enzymatic degradation of OAS-RNase L	Thornbrough et al., 2016
HIV-2	Antagonize tetherin by interacting with viral Rod envelope glycoprotein	Le Tortorec and Neil, 2009
HCV, HIV, IAV, and VACV	E2/NS5A, Tat, NS1, and E3l/K3L viral proteins of respective viruses interact with PKR	Reviewed in Short, 2009

### Penetrating Physical Barriers

Physical barriers, such as skin or the surface of the respiratory, genital, or gastrointestinal tracts, including fluid repleted with antimicrobials, neutralizing immunoglobulins, mucus, and the epithelial cell layers, guard viral invaders. Viruses breach such barriers in a multitude of ways. For instance, specific viral proteins interact with cell receptor proteins present in the apical junctional complex to modify the barrier properties of the epithelium (Gonzalez-Mariscal et al., 2009). Interestingly, lower pathogenic avian influenza viruses generally do not cause severe pneumonia because mucus restrains and removes these viruses before approaching lower respiratory tracts, however, highly pathogenic IAV can breach such mucosal barriers (Van Riel et al., 2010). Although the skin is the most important physical barrier, it contains numerous permissive cells for flavivirus infection, such as ZIKV, DENV, and WNV, therefore, these viruses exploit permissive cells at the first site of infection (Garcia et al., 2017). HIV and SIV appear to be capable of flexibly exploiting multiple mechanisms to transit different epithelial barriers and gain access to susceptible target cells to establish a systemic infection (Keele and Estes, 2011).

### Escaping From the Recognition From PRRs

Knowing that viruses are sensed by PRRs, viruses circumvent/minimize PRRs’ sensing through numerous viral tactics, such as by sequestering/hiding viral genome, interacting with PRRs, targeting/cleaving adapter proteins, and so on. An *in vitro* study determined the effect of HCV proteins (NS3, NS3/4A, NS4B, or NS5A) on the TLR signaling pathways, where cells expressing these proteins were found to inhibit the activation of the TLR2, TLR4, TLR7, and TLR9 signaling pathways (Abe et al., 2007). Several viral proteins can specifically interact with PRRs. For an example, p7 of HCV associates with DNA sensor IFI6-16 (Qi et al., 2017). HBV conceals its genome into the viral capsid to escape away from cGAS sensing (Verrier et al., 2018). The human papillomavirus (HPV) E6 oncoprotein targets USP15 and TRIM25 to suppress RIG-I-mediated innate immune signaling (Chiang et al., 2018). Some viruses have evolved with strategies to modify viral RNAs and viral RNA-binding proteins to escape away from sensing by RIG-I, MDA5 (Bowie and Unterholzner, 2008). Viral dsRNA genome is highly susceptible to recognition by MDA5; both positive-sense RNA viruses and some DNA viruses produce dsRNA intermediate during their replication cycle, while some viruses conceal their dsRNA by encoding dsRNA binding proteins or even sequester viral RNA (Table 1). More interestingly, unlike positive-strand RNA and DNA viruses, negative-sense RNA viruses uniquely do not produce dsRNA intermediates; this unique property minimizes detection by PRRs (Weber et al., 2006). Moreover, a recent report showed that SARS-CoV-2 RNAs are capped at the 5’ end and escape recognition from PRRs (Encinar and Menendez, 2020). Viral mechanisms of escaping from PRRs’ recognition have been reviewed elsewhere (Weber et al., 2006; Abe et al., 2007; Bowie and Unterholzner, 2008; Chan and Gack, 2016; Qi et al., 2017; Chiang et al., 2018; Verrier et al., 2018; Encinar and Menendez, 2020; Kikkert, 2020; Lhomme et al., 2020; Liu et al., 2020; Zhu and Zheng, 2020).

### Inactivation of Transcriptional Factors

At the basal level, transcriptional factors are inactive. After viral infection, transcriptional factors, such as IRF3/5/7, NF-κB, AP1, and others translocate into the nucleus and then induce the robust expression of IFNs (Christensen and Thomsen, 2009; Iwasaki and Pillai, 2014; Chen et al., 2018). Several conserved viral proteins, predominantly non-structural (NS) proteins, are extensively reported to exert potent antagonistic effects of IFN responses by several mechanisms. Viruses are reported to inhibit IFN induction by inducing degradation transcription factors, inhibiting their activation by blocking downstream signaling of PRRs, sequestering them, impeding their nuclear translocation, or inhibiting their binding to promoters of downstream antiviral genes, and so forth (Table 1). For example, IAV NS1 protein, extensively characterized as a potent antagonist of IFN-signaling, inhibits activation and nuclear translocation of IRF3 and NF-κB (Wang et al., 2000). Human cytomegalovirus (HCMV) is well-known for establishing long-term latent infections. The innate immune escape strategy of HCMV is appeared to be pivotal for establishing such infections. UL44 protein of HCMV decelerates antiviral responses by inhibiting the binding of IRF3 and NF-κB to the promoters of downstream antiviral genes (Fu et al., 2019).

### Regulating the Transcriptional and Translation of Key Elements of Innate Immunity

Transcription and translation of key elements of antiviral innate immunity, such as PRRs, IRFs, IFNs, STATs, ISGs, and others are very important for eliciting an antiviral response. Cumulative reports suggest that viruses can deregulate the transcription and translation of such elements. *Caliciviridae*, *Coronaviridae*, *Picornaviridae*, *Orthomyxoviridae*, *Reoviridae*, and many others exploit multiple tactics to induce host translational shut-off and thus prevent the infected cells from synthesizing new peptides and proteins, including those IFN-stimulated IRFs and STATs (reviewed in Chiang and Liu, 2019). HIV-1 Vpu protein potently suppresses NF-κB-elicited antiviral immune responses at the transcriptional level (Langer et al., 2019). Epstein-Barr virus BRLF1 inhibits the transcription of IRF3 and IRF7 (Bentz et al., 2010). IAV induces rapid degradation of eukaryotic translation initiation factor 4B (an integral component of the translation initiation apparatus) and contributes to viral replication at least by suppressing IFITM3 protein expression (Wang et al., 2014).

### Antagonizing IFN Induced JAK-STAT Signaling

Besides the aforementioned viral escape strategies, viruses have evolved strategies to antagonize IFN and its downstream signaling through numerous sophisticated mechanisms. Mechanisms include targeting degradation of IFNs receptors, retention of suppression of STATs in the cytoplasm, inhibition of STAT activation, degradation of STATs through the proteasome, and so forth. NS4B protein of several flaviviruses inhibits IFN signaling-induced JAK-STAT signaling in a multitude of ways (Munoz-Jordán et al., 2005). Flavivirus NS5 protein dysregulates HSP90 to inhibit JAK/STAT signaling (Roby et al., 2020). NS2A, NS2B, NS3, NS4A, and NS4B proteins of WNV block STAT1 and STAT2 activation (Liu et al., 2005). 2A proteinase of Enterovirus 71 degrades IFNAR1 (Lu et al., 2012). HCV and flaviviruses hijack cellular mechanisms for nuclear STAT2 degradation by up-regulation of PDLIM2 (Joyce et al., 2019). Nsp5 protein of porcine deltacoronavirus, an emerging coronavirus, cleaves STAT2 (Zhu et al., 2017). Orf6 of SARS-CoV-2 hijacks Nup98 to block STAT nuclear import (Miorin et al., 2020). There is a range of literature regarding the viral invasion of innate immunity by antagonizing IFN and IFN induced downstream signaling (Liu et al., 2005, 2020; Lu et al., 2012; Chan and Gack, 2016; Scott and Nel, 2016; Zhu et al., 2017; Maarouf et al., 2018; Joyce et al., 2019; Acharya et al., 2020; Miorin et al., 2020).

### Viral Invasion of ISGs

Global ISG response plays a very important role in viral clearance. Delayed ISG production is advantageous for viral replication and spread into host tissues. Viruses also exploit several distinct approaches to antagonize the global ISG response (Short, 2009). Highly pathogenic influenza viruses and coronaviruses induce repressive histone modifications, which downregulates the global expression of ISG subsets (Menachery et al., 2014). Although interferon-induced transmembrane proteins (IFITMs) play an antiviral role against a large group of viruses, particularly during viral invasion to the host at entry stage, human cytomegalovirus can exploit IFITM proteins to facilitate morphogenesis of the virion assembly compartment (Xie et al., 2015). Viral invasion of ISGs has been extensively reviewed elsewhere (Table 1; Kanodia et al., 2007; Short, 2009; Su et al., 2016).

### Regulating Autophagy

Autophagy, an autonomous arm of innate immunity, is a cytosolic lysosome-dependent catabolic process that mediates viral clearance. Autophagy can be upregulated upon virus detection by pathogen receptors, including membrane-bound and cytosolic PRRs, and which may further facilitate PRR-dependent signaling and also contribute induction of type I IFNs (Richetta and Faure, 2013). Beclin-1 is an essential macro-autophagy protein that constitutes part of the phosphatidylinositol-3 kinase complexes that mark membranes for autophagosome generation and facilitate autophagosome fusion with lysosomes (Münz, 2011). α-herpesvirus Akt-like Ser/Thr kinase limits autophagy to stimulate virus replication by inhibition of ULK1 and Beclin1 (Rubio and Mohr, 2019). Pseudorabies virus infection inhibits autophagy in permissive cells *in vitro* (Sun et al., 2017). Viral proteins ICP34.5, orf16, and M11 of viruses HSV-1, KSHV, and MHV-68, respectively, block autophagosome generation, whereas nef and M2 viral proteins of HIV and IAV, respectively, inhibit autophagosome maturation (Choi et al., 2018). Readers can also refer to some previously published review papers (Münz, 2011; Richetta and Faure, 2013; Jackson, 2015; Lennemann and Coyne, 2015; Sun et al., 2017; Choi et al., 2018; Rubio and Mohr, 2019).

### Other Mechanisms

In addition to the aforementioned viral immune escape mechanisms, a large group of viruses often encode proteins to inactivate released cytokines or chemokines by binding, solubilizing, and altering the cellular responsiveness (Lucas et al., 2001). Numerous viruses including HIV-1, HCV, HBV, HSV-1, RSV, EBOV, IAV, and others induce robust expression of suppressors of cytokine signaling (SOCS) proteins (Akhtar and Benveniste, 2011). SOCS proteins induced by cytokine signaling during viral infection function as negative feedback regulators to reduce inflammation and promote viral replication (Akhtar and Benveniste, 2011). More interestingly, some viruses can directly induce SOCS proteins independently of cytokine signaling. For example, the influenza virus induces the expression of SOCS3 in a cytokine-independent manner to circumvent IL-6/STAT3-mediated immune response (Liu et al., 2019). Virus-induced stress granules, the cytoplasmic dense aggregates of proteins and RNAs produced when cells are in stress, can also play an important role in innate immunity by recruiting viral sensors, such as RIG-I, MDA5, PKR, and so forth to initiate downstream antiviral innate immune signaling (McCormick and Khaperskyy, 2017). Several viruses brilliantly inhibit such stress granule formation by diverse mechanisms (Wu et al., 2014; McCormick and Khaperskyy, 2017). Moreover, viruses can also circumvent antiviral immunity through sequestering critical elements of innate immunity, such as TBK1, IKKε, and IRF3 into viral inclusion bodies (Wu et al., 2014). Other famous escape mechanisms include hijacking transcriptional and translational machineries for their survival, which can also mediate the circumvention of innate immune response in multiple ways. For example, the Nsp1 protein of SARS-CoV-2 mediates host translation shutdown and evades innate immunity (Thoms et al., 2020).

## Perspectives and Conclusion

Obstructing viral immune invasion could potentially provide an alternative approach for the prevention and treatment of disease caused by an acute infection of viral pathogens. Increasing data regarding viral innate immune escape mechanisms have been reported. However, most of these data are limited to *in vitro* (cell culture system) and *in vivo* animal models. The relevance of viral escape mechanisms identified by these models may not apply the same in human. Therefore, this issue remains to be addressed by extensive *ex vivo* experiments in the human model. The molecular basis of antiviral innate immune signaling is complex, multi-waved, inter-connected, and may not always be antiviral. For instance, it is well-known that TLR signals induce robust expression of antiviral innate immunity for viral clearance. However, in certain circumstances, the activation of particular TLR responses by pathogens might serve as an escape mechanism from the host defense (Netea et al., 2004). Furthermore, studies for the in-depth understanding of virus-host interaction are very important because the molecular basis of viral escape mechanisms and crosstalk among immune signaling for the progression of disease are still largely unexplored.

Of several conserved viral proteins, predominantly NS proteins appear to be major antagonists of the elements of innate immunity. Since viral NS proteins play a vital role in innate immune escape mechanisms, there is a pressing need for scientists to uncover host factors countering those viral NS proteins. Supportively, recent reports have arguably characterized host factors countering such viral proteins. For instances, virus-induced TRIM22, viperin, and p27Kip1 mediate rapid degradation of HCV NS5A, ZIKA NS, and IAV NS1 proteins, respectively (Yang et al., 2016; Panayiotou et al., 2018; Rai et al., 2020). Identification and characterization of such types of host factors countering these viral proteins in the future are truly indispensable in elaborating antiviral innate immunity.

A virus may exploit numerous and multiple immune escape tactics collectively and cooperatively for effective immune evasion. However, most of the previously published experimental data are mostly limited to viral escape tactics specifically at the individual level. Comprehensive studies on how viruses exploit their overall immune escape tactics together for disease progression or host killing in acute viral infection or in establishing successful infection should have been experimentally substantiated. Moreover, the consequences of cytokine storms in acute viral infection have been widely studied but the mechanistic basis of differential cytokine storm production and why the magnitude of cytokine storm production differs from one individual to another are largely unknown.

In conclusion, an acute viral infection can cause sudden or rapid onset of disease that may be resolved quickly or may be fatal. Innate immunity provides the first line of defense for viral clearance. However, viruses have evolved strategies to escape the host’s antiviral innate immune surveillance that may kill the host or establish persistent infections. There are still many unanswered questions regarding the impact of viral escape strategies on host killing and viral persistence. Comprehensive understanding of the underlying complex molecular basis of viral escapology would help provide landmark achievements in our ongoing battle against viral infections.

## Author Contributions

KR, PS, BY, YC, SL, and MM performed a systematic literature review. KR wrote the manuscript. J-LC organized and critically revised the manuscript. BY helped in the manuscript revision and drawing figure. All authors have read and agreed to the published version of the manuscript.

## Conflict of Interest

The authors declare that the research was conducted in the absence of any commercial or financial relationships that could be construed as a potential conflict of interest.

## Abbreviations

AP-1: Activator protein 1
EV: Extracellular vesicles
HERV: Human endogenous retrovirus
HIV: Human immunodeficiency virus
HSV: Herpes simplex virus
IFITM: Interferon-induced transmembrane protein
IKK: I-kappa -B kinase
KSHV: Kaposi’s sarcoma-associated herpesvirus
MAVS: Mitochondrial antiviral signaling protein
MHV: Mouse hepatitis virus
MX1: Myxovirus Resistance Protein 1
MyD88: Myeloid differentiation primary response 88
NF-kB: Nuclear factor kappa-light-chain-enhancer of activated B cells
RIP1: Receptor interacting protein
SIV: Simian immunodeficiency virus
Syk: Spleen tyrosine kinase
TBK1: TANK-binding kinase 1
TRAF: TNF receptor associated factors
TRIF: TIR-domain-containing adapter-inducing interferon-β.
